# Cladribine treatment specifically affects peripheral blood memory B cell clones and clonal expansion in multiple sclerosis patients

**DOI:** 10.3389/fimmu.2023.1133967

**Published:** 2023-03-07

**Authors:** Christoph Ruschil, Gisela Gabernet, Constanze Louisa Kemmerer, Mohamed Ali Jarboui, Franziska Klose, Sven Poli, Ulf Ziemann, Sven Nahnsen, Markus Christian Kowarik

**Affiliations:** ^1^ Department of Neurology and Stroke, Center for Neurology, Eberhard Karls University of Tübingen, Tübingen, Germany; ^2^ Hertie-Institute for Clinical Brain Research, Eberhard Karls University of Tübingen, Tübingen, Germany; ^3^ Quantitative Biology Center (QBiC), Eberhard Karls University of Tübingen, Tübingen, Germany; ^4^ Core Facility for Medical Bioanalytics (CFMB), Eberhard Karls University of Tübingen, Tübingen, Germany; ^5^ Biomedical Data Science, Department of Computer Science, Eberhard Karls University of Tübingen, Tübingen, Germany

**Keywords:** multiple sclerosis, B cells, cladribine, immunoglobulin repertoire, next generation sequencing, immunoglobulin proteome analysis

## Abstract

**Introduction:**

B cells are acknowledged as crucial players in the pathogenesis of multiple sclerosis (MS). Several disease modifying drugs including cladribine have been shown to exert differential effects on peripheral blood B cell subsets. However, little is known regarding functional changes within the peripheral B cell populations. In this study, we obtained a detailed picture of B cell repertoire changes under cladribine treatment on a combined immunoglobulin (Ig) transcriptome and proteome level.

**Methods:**

We performed next-generation sequencing of Ig heavy chain (IGH) transcripts and Ig mass spectrometry in cladribine-treated patients with relapsing-remitting multiple sclerosis (n = 8) at baseline and after 6 and 12 months of treatment in order to generate Ig transcriptome and Ig peptide libraries. Ig peptides were overlapped with the corresponding IGH transcriptome in order to analyze B cell clones on a combined transcriptome and proteome level.

**Results:**

The analysis of peripheral blood B cell percentages pointed towards a significant decrease of memory B cells and an increase of naive B cells following cladribine therapy. While basic IGH repertoire parameters (e.g. variable heavy chain family usage and Ig subclasses) were only slightly affected by cladribine treatment, a significantly decreased number of clones and significantly lower diversity in the memory subset was noticeable at 6 months following treatment which was sustained at 12 months. When looking at B-cell clones comprising sequences from the different time-points, clones spanning between all three time-points were significantly more frequent than clones including sequences from two time-points. Furthermore, Ig proteome analyses showed that Ig transcriptome specific peptides could mostly be equally aligned to all three time-points pointing towards a proportion of B-cell clones that are maintained during treatment.

**Discussion:**

Our findings suggest that peripheral B cell related treatment effects of cladribine tablets might be exerted through a reduction of possibly disease relevant clones in the memory B cell subset without disrupting the overall clonal composition of B cells. Our results -at least partially- might explain the relatively mild side effects regarding infections and the sustained immune response after vaccinations during treatment. However, exact disease driving B cell subsets and their effects remain unknown and should be addressed in future studies.

## 1 Introduction

During the last decade, B cells have been proven to play an important pathophysiological role in multiple sclerosis (MS) and B cells have more and more entered into the focus of therapeutic approaches (1, 2). Although several targets including recent work on possible Epstein-Barr virus (EBV) antigens have been suggested as potential B cell antigens during MS pathophysiology (3), the exact role of B cells in MS remains inconclusive (4). Besides a primary antigen driven role of B cells in MS it has to be taken into consideration that B cells might also serve as antigen presenting cells, produce proinflammatory cytokines and thus might feed autoimmune circuits in the periphery (5–7). This hypothesis is - to a certain point- supported by studies on B cell migration between the periphery and the central nervous system (CNS) which showed a bidirectional exchange of B cells across the blood-brain barrier and suggested that B cell germinal centers may be active on both sides of the blood-brain barrier (8). Furthermore, CSF B cells are clonally connected with B cells in the meninges, and parenchyma of the CNS (4, 8, 9).

High throughput next generation DNA sequencing of immunoglobulin heavy chain (VH) repertoires (B cell repertoire) is a powerful method to gain deep insights into the pathophysiological role of B cells in autoimmune diseases (10–12). Furthermore, treatment specific effects can be assessed to not only detect quantitative changes of B cell subsets but also qualitative changes in terms of B cell maturation and connectivity. We could recently show that fingolimod and natalizumab treatment exert differential effects on B cell trafficking across the blood-brain-barrier and B cell repertoires in MS patients (13). The effects of immunomodulatory treatments such as mycophenolate mofetil or rituximab have also been studied by B cell repertoire sequencing in systemic lupus erythematodes (12). Cladribine is a purine nucleoside analog that has shown to be an effective treatment for multiple sclerosis patients (14–18). Since cladribine tablets have also been shown to affect total CD19+ B cells during the treatment of MS patients (19–21), we were now interested in the effects of cladribine treatment on peripheral blood B cell repertoires.

The purpose of this study was to provide a detailed longitudinal picture on cladribine associated immunoglobulin repertoire changes in MS patients (n = 8). Since we were interested in functional B cell repertoires including Ig proteomics (22) we performed Ig mRNA sequencing in combination with Ig proteomics. We assessed Ig repertoire analyses at baseline (B) and following 6 months (6M) and 12 months (M12, prior to 3^rd^ cycle) of treatment. At each time point, peripheral blood B cells were sorted into naïve, memory, double-negative (DN) and plasmablast populations. Immunoglobulin heavy chain (IGH) repertoires were generated by next generation mass sequencing and further analyzed for repertoire properties. In addition, we assessed Ig peptides by mass spectroscopy and overlapped Ig peptide fragments with IGH repertoires in order to understand, to what extent Ig repertoires from circulating B cells contribute to the Ig serum fraction.

## 2 Materials and methods

### 2.1 Standard protocol approvals, registrations and patients

Patients with relapsing-remitting multiple sclerosis (**Table 1**) were recruited at the neuroimmunological outpatients clinics of the Eberhardt Karls Universität Tübingen. All patients provided written consent to the scientific use of their biologic samples. The study was approved by the local ethics committee of the Eberhardt Karls Universität Tübingen (No. 204/2018BO2).

**Table 1:** Patients characteristics.

ID	Sex	Age baseline	EDSS baseline	EDSS after 6 months	EDSS after 12 months	Date study inclusion (day-mon-year)	Date of diagnosis (month-year)	CSF status (OCB) at diagnosis	First reported relapse (year)	Previous therapy
CLAD1	Female	36	3.5	3.0	2.5	07-Feb-2018	uk-2010	positive	2010	06/12 - 12/12 GLAT 11/13 - 12/17 FTY
CLAD2	Female	39	2.0	2.0	3.5	10-Aug-2018	uk-1996	unknown	1994	uk/98 - uk/02 IFNβ-1a uk/04 - uk/06 GLAT uk/06 - 01/14 AZA 03/14 - 04/18 FTY
CLAD3	Female	29	3.5	3.5	3.5	27-Nov-2018	uk-2007	unknown	2007	uk/07 - uk/09 IFNβ-1a 03/12 - 06/12 INFβ-1a 06/12 - 06/14 NAT 06/14 - 12/16 DMF 02/17 - 09/17 FTY 12/17 - 05/18 NAT
CLAD4	Female	23	2.0	2.0	2.0	14-Jan-2019	Sep-2014	positive	2014	11/14 - 11/18 DMF
CLAD5	Female	24	2.0	2.5	2.5	13-May-2019	uk-2016	positive	2016	08/18 - 04/19 DMF
CLAD6	Female	29	2.0	1.0	1.0	18-Jun-2019	May-2018	negative	2017	08/18 - 05/19 GLAT
CLAD7	Female	27	1.5	1.5	1.5	02-Jul-2019	Apr-2016	positive	2016	09/16 - 05/19 PegIFN
CLAD8	Male	21	2.0	1.5	1.5	08-Jul-2019	May-2018	positive	2018	None

uk, unknown; GLAT, glatiramer acetate; FTY, fingolimod; IFNβ-1a, interferon beta-1a; AZA, azathioprine; NAT, natalizumab; DMF, dimethyl fumarate; PegIFN, Pegylated interferon-beta-1a; EDSS, expanded disability status scale; OCB, oligoclonal bands.

Study inclusion criteria for this single-arm observational study were 1) diagnosis of clinically definite MS according to revised McDonald Criteria (2017); 2) age between 18-65 years; 3) the ability to give informed consent; and 4) normal vital signs. Exclusion criteria were 1) CNS disease in addition to MS; 2) primary progressive or secondary progressive forms of MS; 3) bacterial or viral infection within the last 30 days; 4) prior immunomodulatory or immunosuppressant therapy with methotrexate, cyclophosphamide, mitoxantrone, rituximab, ocrelizumab, daclizumab, mycophenolate mofetil, laquinimod, or total body irradiation; and 5) treatment within the last 30 days with corticosteroids, beta-interferon, glatiramer acetate, plasma exchange, or intravenous immunoglobulin. In case of switching from prior immunomodulatory therapies, at least 30 days of washout, in case of fingolimod or natalizumab 12 weeks and additional normalization of differential blood count was required. For all other previous therapies (such as azathioprine) an interval of at least 3 years and additional normalization of differential blood count was mandatory.

### 2.2 Cell staining and sorting of CSF and blood B cell populations

Peripheral blood (25ml, EDTA blood, 7.5ml serum) was collected from all patients and peripheral blood mononuclear cells (PBMCs, using a Ficoll gradient protocol, stored in liquid nitrogen) and serum was processed within two hours. For flow cytometric analyses, the following antibodies were used for all analyses: CD38 FITC (BD), IgD PE (Biozol), CD19 ECD (Beckman Coulter), CD3 PeCy7 (Beckman Coulter), CD45 V450 (BD), CD27 APC (BD), CD20 APC Cy7 (BD). Fluorescence minus one (FMO) controls were applied to verify gating strategy (gating strategy described previously (23) and shown in **Supplementary Figure S1**). Shortly, B cell subsets were defined by the following markers: naïve B cells CD19 + CD20 + CD27 - CD38 -/low IgD +, memory B cells CD19 + CD20 + CD27 + CD38 low, double negative B cells (DN B cells) CD19 + CD20-/low CD27 - IgD -, plasmablasts CD19 + CD20 -/low CD27 + CD38 high IgD -).

### 2.3 Blood VH transcriptome library preparation and deep sequencing

Next generation sequencing of Ig heavy chain transcripts was performed as described previously (7, 12). Shortly, collected PBMCs were thawed at 37°C, washed in phosphate buffered saline (PBS) containing 2% fetal calf serum (FCS) and incubated with the aforementioned antibodies (40min at 4°C). After another washing step, B cell subtypes were bulk sorted on a FACSAriaIII (BD bioscience) and collected in PBS directly followed by an RNA extraction step (Macherey Nagel NucleoSpin RNA Plus XS, manufacturer’s instructions, RNA stored at -80°C).

After cell sorting and RNA extraction, cDNA synthesis was done using the Clontech SMARTer Ultra Low RNA Kit for Illumina sequencing according to the manufacturer’s instructions. We adopted the second strand synthesis step by additionally adding constant region primers for IgA, IgG, IgM and IgD. After cDNA synthesis, a pool of VH-family-specific (VH1-VH5) and isotype-specific (IgD, IgM, IgG, and IgA) primers were used to amplify VH-region sequences using polymerase chain reaction (PCR high fidelity, Roche); separate PCR reactions for each VH-family were performed to avoid cross-priming/primer competition. Ig constant region primers contained a sequence tag (“barcode”) to identify the cell population of origin. A primer sequence containing unique molecular identifiers (UMI) for subsequent Illumina MiSeq sequencing was included in the PCR primers, to control for sequence duplicates coming from the PCR amplification step. Amplified cDNA from the peripheral blood of each subject at each time point was then pooled and sequenced in a single run on an Illumina MiSeq Personal Sequencer.

Sequencing data was further processed using the nf-core/airrflow pipeline version 2.0.0 (https://doi.org/10.5281/zenodo.2642009), which is an open source workflow written in Nextflow (24) and available at http://github.com/nf-core/airrflow as part of the nf-core project (25). The pipeline employs the Immcantation toolset for processing of repertoire sequencing data. The sequencing quality of the Illumina MiSeq high-throughput sequencing reads was evaluated with FastQC. The pRESTO toolset (26) was used for processing the sequencing reads. Reads were filtered according to base quality (quality score threshold of 20), the forward and reverse reads were paired and a consensus sequence from reads with the same UMI barcodes was obtained, allowing a maximum mismatch error rate of 0.1 per read group. V(D)J sequences were only considered that had at least 2 representative sequences to build the consensus. Sequence copies were calculated as the number of identical sequences with different UMI barcodes.

Variable-diversity-joining [V(D)J] germline segments were assigned by blasting the processed sequences to the IMGT database with IgBLAST (27). Functional V(D)J sequences were assigned into clones based on (1) identical nucleic acid complementarity determining region-3 (CDR3) sequence length, (2) same IGH variable gene segment and IGH joining gene segment and (3) 88% identity of CDR3 nucleotide sequence with the Change-O tool (28). Repertoire characterization was performed by the use of Alakazam (8) and SHazaM (28), respectively. A series of custom scripts were employed to generate clonal overlaps and the paper figures which can be found under https://github.com/qbic-projects/MS-cladribine-study.

### 2.4 Ig peptides libraries recovered by mass spectrometry

For each timepoint additional serum samples were collected and Ig proteome peptides analyzed. Serum (0.5 to 2.0 ml) was applied to Captureselect kappa/lambda columns (Thermofisher) and purified according to the manufacturer’s instructions. Total protein amounts from each patient sample were quantified using the BCA assay quantification kit (Pierce™ BCA Protein Assay Kit). Similar amount of total proteins from each patient were subsequently used for enzymatic digestion with trypsin and processed for LC-MS/MS analysis as described (29). Briefly, trypsin digestion was carried out in 2M Urea, 50mM Tris-HCl pH 7.5, 1mM DTT containing 5μg/ml modified sequencing-grade trypsin (Promega) overnight at 28°C. Peptides were alkylated using iodoacetamide (5mg/ml) and incubated in the dark for 30 minutes at room temperature. Samples were desalted using C18 Stage Tips (Thermofisher) and analyzed by mass spectrometry.

Mass spectrometry analysis was performed on an Ultimate3000 RSLC system coupled to an Orbitrap Fusion Tribrid mass spectrometer (Thermo Fisher Scientific). Tryptic peptides were loaded onto a µPAC Trapping column with pillar diameter 5µm, inter pillar distance 2.5µm, pillar length/bed depth 18µm, external porosity 9%, bed channel width of 2mm and a length of 10 mm, pillars are superficially porous with a porous shell thickness of 300nm and pore sizes in the order of 100 to 200 Å at a flowrate 10µl/min in 0.1% trifluoroacetic acid in HPLC grade water. Peptides were eluted and separated on the PharmaFluidics µPAC nano-LC column – 50 cm µPAC C18 with a pillar diameter of 5µm, inter pillar distance 2.5µm, pillar length/bed depth 18µm, external porosity 59%, bed channel width 315µm and a bad length 50cm, pillars are superficially porous with a porous shell thickness of 300nm and pore sizes in the order of 100 to 200 Å by a linear gradient from 2% to 30% of buffer B (80% acetonitrile and 0.08% formic acid in HPLC-grade water) in buffer A (2% acetonitrile and 0.1% formic acid in HPLC-grade water) at a flow rate of 300nl/minute. Remaining peptides were eluted by a short gradient from 30% to 95% buffer B, the total gradient run was 120 min. LC-MS parameters were as follows: for full LC-MS spectra, the scan range was 335–1,500 with a resolution of 120,000 at m/z=200. LC-MS/MS acquisition was performed in top speed mode with 3 seconds cycle time. The maximum injection time was 50 msec. The AGC target was set to 400,000, and the isolation window was 1.6 m/z. Positive Ions with charge states 2-7 were sequentially fragmented by higher energy collisional dissociation. The dynamic exclusion duration was set to 60 seconds and the lock mass option was activated and set to a background signal with a mass of 445.12002.

LC-MS Data was performed using the MaxQuant software (30) (version 2.0.3). Trypsin was selected as the digesting enzyme with maximal 2 missed cleavages. Cysteine carbamidomethylation was set for fixed modifications, oxidation of methionine and deamidation of asparagine and glutamine were specified as variable modifications. The first search peptide tolerance was set to 20, the main search peptide tolerance to 5ppm. For peptide and protein identification individual databases for each patient obtained from Ig transcriptome sequences were used. A minimum peptide number of 1 and a minimum length of 6 amino acids was tolerated. iBAQ quantification method was used. The match between run option was enabled with a match time window of 0.7 min and an alignment time window of 20 min. For Maxquant identification search, protein and peptide spectral match FDR were set to a minimum of 0.01. The peptide matches to the BCR transcriptome libraries were further filtered to exclude peptides matching to multiple different BCR sequences. Peptides with matches to CDR regions with at least 3 amino-acids were selected. The peptide match filtering analysis, and proteome-transcriptome overlap were performed with custom scripts that can be found under https://github.com/qbic-projects/MS-cladribine-study.

### 2.5 Statistics

For analysis of B cell subset percentages in the peripheral blood the Friedmann test with Dunn’s multiple comparisons was applied using GraphPadPrism V9.1.2. For NGS sequencing results, the Kruskal-Wallis multi-group comparison test was applied to check for significant differences between the different groups using R scripts. In the following the Wilcoxon paired pairwise test was performed if Kruskal-Wallis tests were significant.

## 3 Results

### 3.1 Patient characteristics and clinical data

Eight patients with the diagnosis of multiple sclerosis were recruited for participation based on inclusion/exclusion criteria and signed informed consent. MS treatment with cladribine tablets was pre-determined by the patient’s treating physician. Patient characteristics including age, duration of disease and - if applicable - previous treatments are shown in **Table 1**. All patients had recent clinical disease activity (except patient 7) and/or radiological disease activity (all except patients 5 and 6) within the last year prior to initiation of treatment with cladribine. After initiation of treatment, both clinical and MRI follow-up was grossly stable, with two patients reporting mild clinical relapses that had no corresponding MRI finding, one patient showing worsening in EDSS without any clinical relapse or new or enhancing MRI lesion and three patients with minor progression in MRI but no clinical relapse and stable (or even improving) EDSS. No major infections or other adverse events were reported.

### 3.2 B cell subsets, Ig repertoire sequencing and basic repertoire properties following cladribine treatment

**Figure 1:**
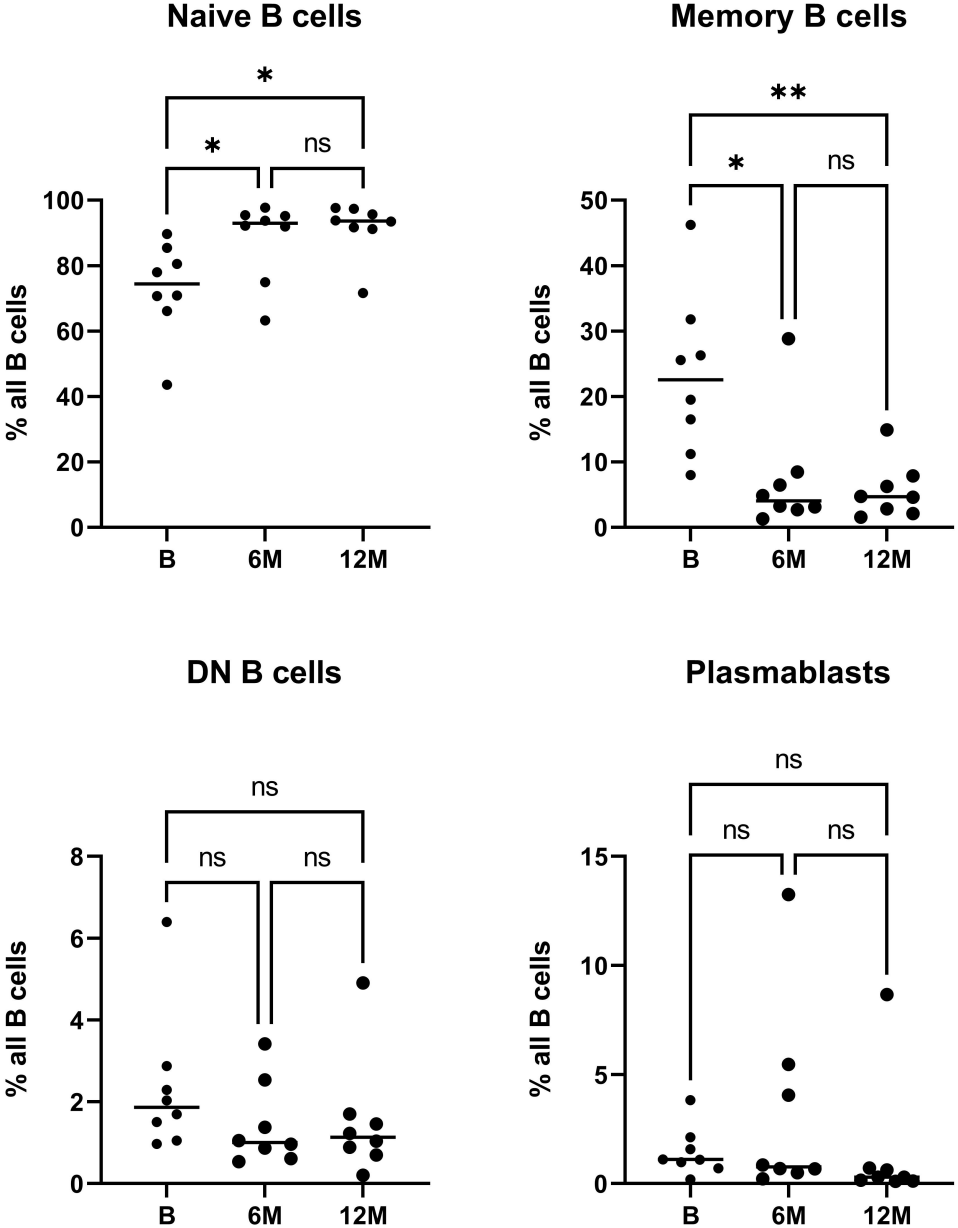
Flow cytometric analysis of peripheral blood B cell subsets during cladribine treatment. The relative frequency of naive, memory, DN B cells and plasmablasts prior to initiation of treatment with cladribine (B) and 6 months (6M) and 12 months (12M) after 1st cycle is shown. Asterisk denote significant (*p<0.05; **p<0.01) differences on Friedmann test with Dunn’s multiple comparisons, ns denotes not significant comparisons.

Distinct treatment-specific changes were noted in the quantitative analysis of peripheral blood B cell subsets (**Figure 1**). For cell sorting, we aimed at always sorting similar amounts of CD45+CD3- cells per patient despite the treatment specific reduction of lymphocytes, in order to assess representative B cell repertoires for each population, especially at 6 months after cladribine application when a significant reduction of immune cells is expectable. Due to the known treatment effects of cladribine on T cells, we excluded this population and -from a practical viewpoint- chose the CD45+CD3- gate including B cells, NK cells and monocytes. Since the latter two populations are less affected by cladribine treatment (21, 31) we did include these populations in order to achieve stable sorting results. When looking at the percentage distribution of B cells following cladribine treatment, a significantly increased percentage of naive B cells after 6 and 12 months was detectable while the percentage of memory B cells was significantly decreased at both time-points (**Figure 1**). A slight decrease of plasmablasts and especially DN B cells was also noticeable, however, differences did not reach significance possibly due to low patient numbers. No correlation between the number of previous treatments and the distribution of B cell subsets was found. In addition, we did not observe a correlation between age (baseline) and the distribution of B cell subsets.

We successfully generated Ig repertoires for all 8 patients, except for one patient (CLAD4) in which we failed to generate a sufficient number of sequences for one population (naive) at one time-point (baseline, only 135 sequences detected) and therefore had to be excluded from further analysis. The average number of sorted B cells was 211193 (range 80298 - 308219) cells at baseline, 181982 (range 64972 - 425004) cells at 6 months and 327131 (range 54645 - 1017071) cells at 12 months. The average number of recovered VH sequences was 38503 (range 22691 - 62545) at baseline, 40185 (range 27474 - 76794) at 6 months and 41231 (range 24870 - 79131) at 12 months (further details are shown in **Table 2**).

**Table 2:** Sorted B cells and recovery Ig sequences for each patient and time-point.

	Baseline			6 months			12 months		
	Sorted B cells	Unique sequences with 2 representatives	Number of sequences after IgBlast	Sorted B cells	Unique sequences with 2 representatives	Number of sequences after IgBlast	Sorted B cells	Unique sequences with 2 representatives	Number of sequences after IgBlast
CLAD1	193991	27529	22691	268127	37554	31930	581877	61332	42056
CLAD2	208036	30760	27239	425004	72975	52052	1017071	61944	44995
CLAD3	280632	67212	50407	179550	58008	37343	102497	57953	39613
CLAD4	80298	44866	35761	78145	40667	32298	54645	35811	24870
CLAD5	308219	48188	30648	194339	42596	27474	251382	40801	27025
CLAD6	200612	72155	62545	138083	91769	76794	359947	93580	79131
CLAD7	123159	61585	47015	64974	47139	30738	125506	53373	40070
CLAD8	294599	51004	31714	107631	44271	32852	124121	49833	32090

When looking at the number of recovered VH sequences for each B cell subpopulation and time-point (B = baseline, 6M = 6 months, 12M = 12 months), no significant effects on the total number of repertoire sequences could be observed for each B cell subtype most likely due to our sorting approach.

Regarding the VH germline distribution, significant effects were noticeable in the naive B cell subset; while a significantly lower usage was observed for IGHV1 when comparing 12M to B and IGHV5 when comparing 12M to 6M and B, a significantly increased usage for IGHV3 segments was observed when comparing 12M to 6M. No significant changes were observed for the other B cells subsets (**Supplementary Figure S4**). For VH isotype frequencies, significantly more IgM isotypes were observed in DN B cell population when comparing 12M to B; no significant effects were observed for the other B cell subsets (**Supplementary Figure S5**).

### 3.3 Cladribine treatment specifically alters memory B cell repertoire properties

To also assess qualitative changes within the Ig repertoires after cladribine treatment, we analyzed B cell clones at each time-point and between the different time-points for each B cell population.

**Figure 2:**
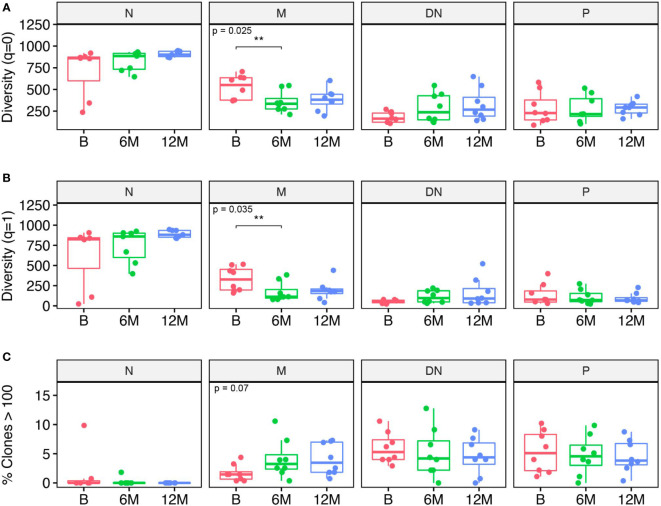
Clonal analysis of B cell repertoires for each B cell subset including naive (N), memory (M), double negative (DN) B cells and in plasmablasts (P) at baseline (B), 6 months (6M) and 12 months (12M) following cladribine treatment. **(A)** Clonal diversity expressed as Hill numbers for q=0. This corresponds to the total number of clones for each B cell subset and time-point. **(B)** Clonal diversity expressed as Hill numbers for q=1, which takes into account the number of clones and their proportions in the repertoire. **(C)** Percentage of clones comprising more than 100 retrieved sequences/clone. Diversity values were obtained on a bootstrap sample of n = 951 sequences with 200 repetitions. P values show Kruskal-Wallis multi-group comparison tests. Asterisks denote Wilcoxon Rank-Sum paired tests performed if Kruskal-Wallis tests were significant (**p<0.01).

When looking at the total number of B cell clones at each individual time-point, numbers were significantly reduced in the memory subset after 6 months of treatment when compared to baseline (**Figure 2A**). A similar effect was noticeable when analyzing clonal diversity weighted by clonal size; again, a significantly decreased diversity was observed for the memory B cell subset when comparing 6 months after treatment with baseline (**Figure 2B**). Although the number of clones seem to slightly increase after treatment in the DN B cell population, no significant differences could be observed for the other B cell populations and time-points. We also looked at the number of clones with more than 100 contributing sequences and found a tendency (p = 0.07) towards an increase in the number of these “bigger” clones in the memory subset after 6 months when compared to baseline whereas no effects were observed for the other subsets and time-points (**Figure 2C**). Altogether, cladribine treatment significantly reduced the absolute number of clones in the memory B cell subsets while the remaining clones seemed to be highly expanded.

**Figure 3:**
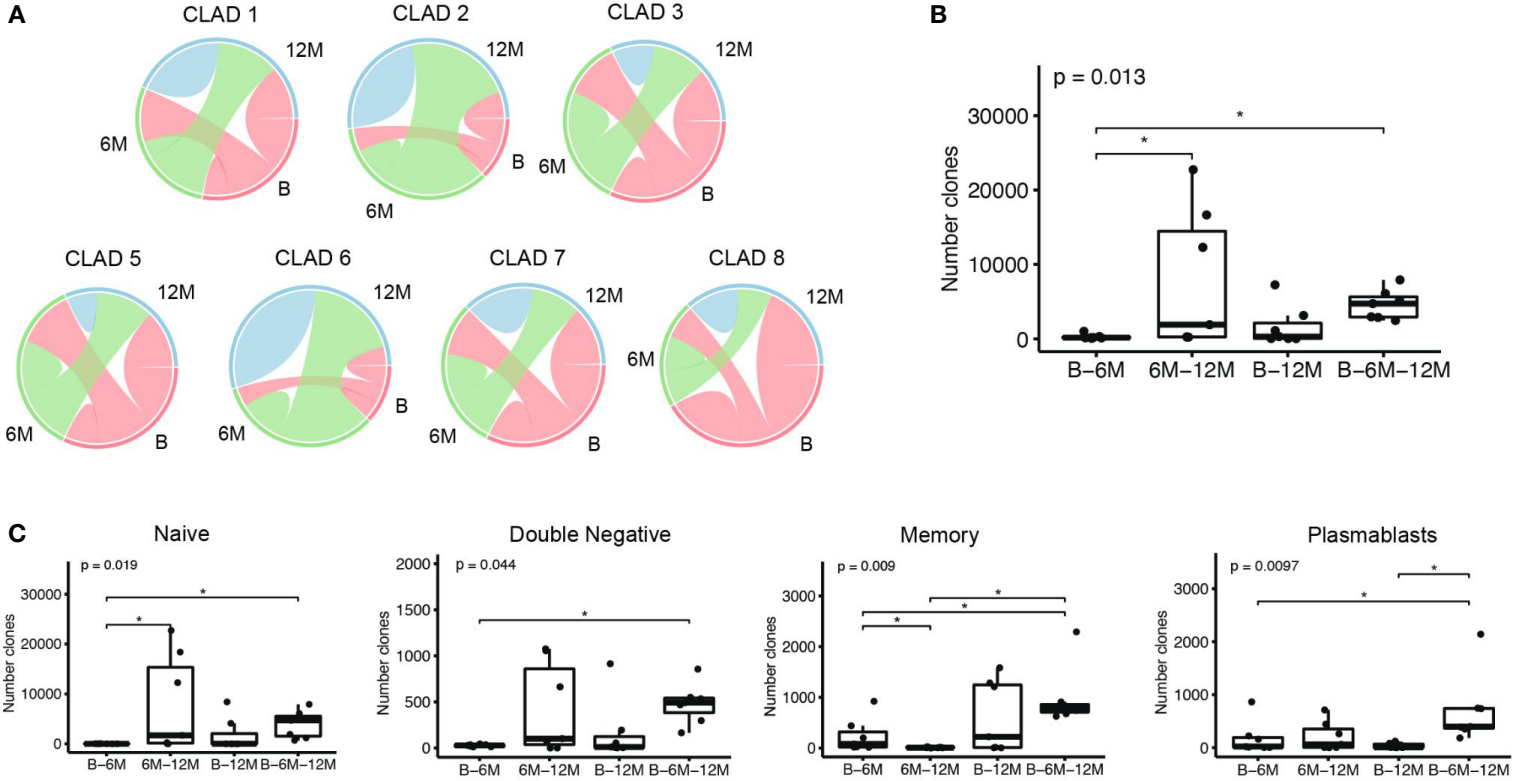
Clonal overlap analyses among the different time-points: baseline (B), 6 months (6M) and 12 months (12M) following cladribine treatment. **(A)** Overlap plots show the proportion of B-cell clones comprising sequences that span over the different time points, and the proportion of clones comprising sequences that are only present in that time point; CLAD4 was excluded from this analysis due to an insufficient sequencing of the naive population. **(B)** Boxplots show the number of clones that comprise sequences of the defined time points (x axis). **(C)** Boxplots show the number of clones that comprise sequences at the defined time points (x axis), when considering the B cell subsets individually. P values show Kruskal-Wallis multi-group comparison tests. Asterisks denote Wilcoxon rank-sum paired tests performed if Kruskal-Wallis tests were significant (*p<0.05).

Besides the clonal diversity at each individual time-point, we also looked at the clonal overlap between the different B cell subsets and time-points (**Figure 3**). The overall clonal connectivity -including all B cell subsets- between the different time-points did not show significant changes and seemed to be maintained during cladribine treatment (including all clones across all time-points, **Figure 3A**). In order to obtain a more complete picture we further looked at clones between B - 6M, 6M -12M, B – 12M or B – 6M – 12M that were exclusively found between these time points (**Figure 3B**). Around 60% of the clones could be related to all three timepoints whereas only 2% connected between B - 6M, 12% between baseline - 12M and 26% between 6M - 12M; significantly more clones were found between 6M - 12M as well as B - 6M - 12 when compared to B – 6M. This indicates that a substantial proportion of clones is preserved during treatment and that newly evolving clones increase between 6M – 12M. When looking at the clonal distribution between time points on a B cell subset level (**Figure 3C**), significantly more clones were observed when comparing clones between B - 6M - 12M and B - 6M for all subsets. However, significantly less clones between 6M - 12M when compared to clones between B - 6M and B - 6M - 12M were observed in the memory population only pointing towards a more sustained effect on clonal development in the memory subset. For the naive B cell subset, significantly more clones were found between 6M - 12M when compared to B - 6M. In the plasmablast population, significantly more clones between B - 6M - 12M were observed when compared to clones between B - 12M.

### 3.4 Cladribine effects on Ig proteome

**Figure 4:**
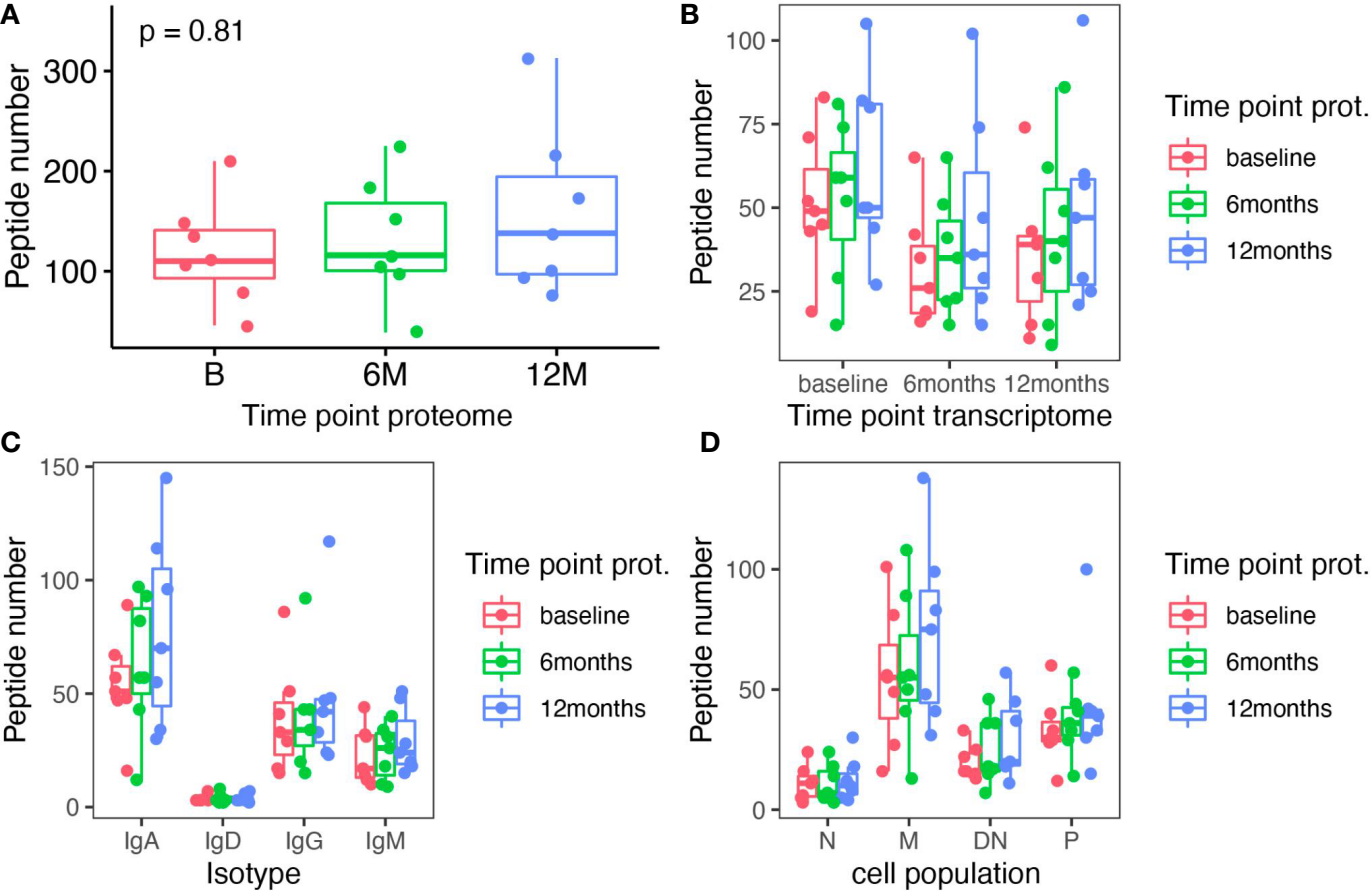
Analysis of Ig peptides that specifically aligned to Ig transcriptome sequences in each individual patient. **(A)** The total number of specific Ig peptides recovered at each time-point (baseline (B), 6 months (6M) and 12 months (12M) following cladribine treatment) is shown. **(B)** Specific Ig peptides from each time point (color coded) are plotted against the matching Ig transcriptome sequence (x axis) from each time-point. **(C)** Specific Ig peptides from each time point (color coded) are plotted against the matching Ig transcriptome sequence sorted by isotypes (x axis) from each time-point. **(D)** Specific Ig peptides from each time point (color coded) are plotted against the matching Ig transcriptome sequence sorted according to B cell subsets (x axis) from each time-point. None of the differences between the different time points reached significance (Kruskal Wallis test).

In order to obtain a comprehensive picture of cladribine related effects on the humoral immune response, we also analyzed the Ig peptide libraries obtained in parallel to the Ig transcriptome data. By aligning Ig peptide fragments to specific regions (CDR regions) of each individual patient’s VH transcriptome, we determined treatment effects on the circulating Ig proteomics. On average, we were able to identify 623 specific peptides (range 458 - 1152) per patient across all time-points. For each time-point a relatively stable number of specific Ig peptides could be aligned (no significant differences) to the VH transcriptome (**Figure 4A**). Based on the Ig peptides libraries from each time-point, we also assigned the matching VH sequences from each individual time-point and found a relatively stable proportion of attributable VH sequences; although slightly more peptides could be assigned to the baseline transcriptomes, differences did not reach significance (**Figure 4B**). We also analyzed whether one Ig peptide could specifically be aligned to a VH sequence from B, 6M or 12M only, the combination of two or all three time points. For the majority of recovered peptides (37%), an Ig peptide could be aligned to VH sequences present at all time-points, whereas matching VH sequences at two time-points or one time-point were less frequently found; a slightly increased proportion of matching VH sequences was found at baseline and 12M and 12M alone. In line with the results from our clonal analysis of VH transcriptome repertoires, a considerable amount of stable B cell clones seems to be maintained throughout the treatment with cladribine tablets. No significant effects between the different timepoints were observed when analyzing the specific Ig peptides from each time-point towards their alignment to the different Ig transcriptome isotypes or Ig transcriptome B cell subsets (**Figures 4C, D**).

## 4 Discussion

B cells play an important role during MS pathophysiology but their exact function, especially of peripheral blood B cells, still remains inconclusive. In this study we were interested in the effects of cladribine treatment on peripheral blood B cells not only in regard to quantitative changes of different B cell subsets but also qualitative changes of B cell repertoires on a transcriptome and proteome level. Since cladribine tablets are applied as an impulse therapy and lymphocytes repopulate during the first year of treatment, we analyzed B cell repertoire changes at baseline (B), after 6 months (6M) and after 12 months (12M) before re-treatment in order to understand, which B cell subset is mainly affected and might possibly drive MS pathophysiology.

Although our analysis was limited by patient numbers, our study provides detailed longitudinal information on the differential treatment effects of cladribine tablets on B cell repertoires. Regarding the quantitative changes of peripheral blood B cell subsets, we found a relative reduction of memory B cells and relative increase of naive B cells while other B cell subsets were not significantly affected; however, a slightly increased percentage of DN B cells was noticeable. Qualitative analyses of our VH repertoires revealed that cladribine treatment only had minor effects on VH germline usage in the naive B cell subset and an increased IgM isotype usage in the DN B cell subsets after cladribine treatment while no significant effects were found for these parameters in the other B cell subsets. Detailed analyses of B cell clones within the VH repertoires showed that the total number of clones and the clonal diversity was significantly reduced in the memory B cell population. Furthermore, the remaining clones within the memory B cell subset were relatively big in size and clonally expanded, indicating that a proportion of memory B cell clones is still present after the first two cladribine applications while the overall number of clones is significantly diminished. In contrast, the number of clones and clonal diversity of the other B cell subsets were not significantly altered.

When looking at the overall clonal connectivity between baseline, 6 months and 12 months, the number of overlapping clones did not change significantly during treatment. Further analyses on the exclusive distribution of clones between certain time-points revealed significantly more clones spanning between B - T6 - T12 and T6 - T12 when compared to clones between B - T6 with the majority of overlapping clones (around 60%) found at all three time points. These data suggest that a proportion of clones is maintained among all B cells and a certain amount of new clones might emerge 6 months after treatment initiation. Although a certain proportion of clones is also maintained across all three timepoints in the memory B cell population, emerging new clones between 6M - 12M seem to be more affected by cladribine treatment, again pointing towards a specific effect of cladribine tablets on the memory B cell pool. Regarding patient specific Ig proteomes, we observed a consistent specific overlap between Ig peptides and Ig transcriptomes across all three time points indicating that a substantial proportion of B cell clones is maintained during cladribine treatment and remains functionally intact by the secretion of the corresponding antibodies. Altogether, our data indicate that on the one hand the overall peripheral blood B cell repertoire remains functional active with a significant amount of clones across the different B cell populations defying cladribine treatment. On the other hand, profound effects could be detected for the memory B cell population with a significant reduction of clones and disruption of newly arising clones.

Our results on peripheral blood B cell percentages are in line with previous flow-cytometric analyses in peripheral blood B cell which showed a reduced memory B cell fraction following cladribine treatment (32–34). Alemtuzumab, which is also administered as a pulsed therapy, has been shown to exert similar effects on peripheral blood memory B cells (35). In addition, we could show that multiple treatments including dimethyl fumarate, interferon beta and fingolimod all lead to a significantly decreased peripheral blood memory B cell fraction (36) pointing at a pathophysiologic role of memory B cells in MS. An overall pathophysiological role of peripheral blood B cells is strongly supported by the high efficacy of peripheral B cell depleting anti CD20 antibodies that are supposed to enter the CNS in limited concentrations (35, 37). Given the strong evidence for an intrathecal B cell activation and expansion (4), it was surprising that intrathecal rituximab treatment did not show substantial better effects than peripheral B cell depletion (37). However, it has to be noted that intrathecal effects of rituximab might be hampered by the lack of intrathecal complement and the consecutively limited effects regarding B cell depletion. Only very limited data is available regarding B cell repertoire changes under different MS medications. In previous work, we could reveal treatment specific effects on peripheral B cell repertoires after initiation of fingolimod or natalizumab treatment although we were mainly interested in effects of both treatments on B cell trafficking across the blood-brain-barrier in this study. B cell receptor sequencing during fingolimod treatment revealed a reduced average number of clones but increased number of average sequences whereas the number of clones and average sequences remained stable under natalizumab treatment (13). However, it has to be noted that natalizumab treatment blocks migration of B cells across the blood-brain-barrier for which reason the composition of peripheral blood Ig repertoires does not allow direct conclusions on MS pathophysiology (13). When summarizing the effects of different MS treatments on peripheral blood B cells, the reduction or depletion of peripheral B cells and B cell clones seem to have a profound impact on MS disease activity possibly by altering mechanisms such as antigen presentation and cell-to-cell communication. Taken together, our findings on cladribine related effects further support the potential disease driving role of peripheral B cells and especially memory B cells in MS since cladribine treatment specifically reduced the number of circulating memory B cells, the clonal diversity of memory B cells and their potential for clonal expansion.

The mechanistic background for a cladribine dependent depletion of peripheral blood lymphocytes has been well established. Cladribine is a synthetic deoxyadenosine analog prodrug, which preferentially depletes cells with high intracellular ratios of deoxycytidine kinase to 5’-nucleotidases which leads to the accumulation of cladribine-phosphate in these cells. Cladribine-phosphates interfere with DNA synthesis and repair and consecutively lead to cell death (38). Although the exact mechanisms for a specific effect on memory B cells are unknown yet, this mode of action coupled with generally low repopulation kinetics of memory B cells might explain why memory B populations are especially vulnerable to cladribine (32).

When relating our sequencing data to the clinical disease course of our patients, no direct correlations can be established since our patient cohort was mostly stable during the first year. However, data from phase III studies revealed that cladribine efficiency increases after the 2nd year of treatment (39). Given that a subset of B cell clones of pathogenic potential within the memory B cell pool or other B cell subsets might be depleted after the first two applications (in the first year) whereas certain B cell clones still persist, one could argue that the repeated administration of cladribine tablets (in the second year and possibly beyond) might additionally deplete pathogenic B cell clones. On the flipside, especially in times of the recent SARS-CoV-2 pandemic, side effects and especially infectious diseases have to be taken into consideration during MS treatments. In this context our data show that naive, DN B cells and plasmablasts B cell repertoires are only slightly affected by cladribine treatment, the clonal connectivity between the different populations remains intact and a substantial proportion of clones persists among the B cell subsets and consistently overlaps with the Ig proteome. Altogether these data point towards a functional B cell response during cladribine treatment which is supported by the relatively mild side effects and effective vaccination against SARS-CoV-2 (40). An efficient antibody response was also observed in cladribine treated MS patients receiving seasonal influenza and varicella zoster virus vaccinations (41). The unique repopulation kinetics and rapid recovery of immature B cells as observed in our study could explain the specific and sufficient B cell response under cladribine treatment. Compared to other pulsed therapies such as alemtuzumab, cladribine treatment has so far not been associated with other auto-immune disorders occurring during repopulation of peripheral immune cells. This effect could also be explained by our data that showed significant effects on the memory B cell subset while the other subsets remain relatively stable.

Although we were able to generate representative repertoires in all our patients, some limitations of our study have to be discussed. Overall, next generation sequencing still seems to be the method of choice to assess huge repertoires of bulk sorted B cells, however, Ig repertoire analyses only allow a snapshot of the B cell repertoires at a certain time and do not fully represent the dynamic relationship amongst B cell subsets. Furthermore, results might be skewed by the process of repertoire reconstruction as sequencing efficiency is different between the populations and despite safeguards such as high-fidelity PCR and UMIs, sequencing errors and over-amplification of transcripts (especially in DN B cells and plasmablasts) may result in artificial, or over-represented B cell clones. Although we aimed at sorting similar amounts of B cells to representatively assess B cell repertoires and clonal relationships within each B cell population, our sorting approach might still have been biased at initial sorting steps due to differential changes of immune cell populations. Our results might thus be hampered by lower frequencies of memory B cells to be sequenced during treatment. Another confounding factor might include different activation states between B cell subsets and time-points which might lead to different amounts of Ig mRNA/per cell and consequently a different sequencing efficiency. From a clinical perspective, our patient cohort was small and biased towards a rather active collective prior to study inclusion. In addition most of the patients (7/8) were previously treated with (multiple) other medications and most of the patients were female (7/8), hence this study might not be representative for previously untreated or male patients. Nevertheless, we were able to assess representative Ig transcriptome and proteome libraries from all patients and observed consistent results from our cohort. Due to the limited sample size and the high activity at baseline, it was not possible to differentiate between responders and non-responders to cladribine tablets during the first year of treatment. Although a mild disease activity was observed in some patients, an overall stable disease course was noticeable during the first year of treatment in our patient cohort.

In conclusion our study shows that cladribine treatment might significantly affect the memory B cell subset, not only quantitatively but also qualitatively by a significant reduction of B cell clones which points out a possible pathophysiologic role of peripheral memory B cells in MS. However, the overall clonal composition of the other B cell subsets and the clonal connectivity between the B cell subsets was only slightly affected in our study, potentially explaining relatively mild side effects and the good vaccination response against SARS-CoV-2. Since exact pathophysiological relevant B cell subsets are currently unknown in MS, we could not analyze the repertoires towards a specific depletion of pathogenic B cell subsets. Future studies are necessary to further dissect disease driving B cells in MS and to specifically deplete these B cell subsets, possibly within the peripheral blood memory B cell subset.

## Data availability statement

The data presented in the study are deposited in the SRA repository, accession number PRJNA906479.

## Ethics statement

The studies involving human participants were reviewed and approved by Ethics committee of the Eberhardt Karls Universität Tübingen. The patients/participants provided their written informed consent to participate in this study.

## Author contributions

CR treated the patients, collected the patient samples, performed transcriptome library preparation and Ig peptide preparation, analyzed the data, did statistical analysis, did literature research, wrote the manuscript, critically reviewed and revised the manuscript. GG analyzed the data, performed statistical analysis, curated the data and developed bioinformatics for transcriptome and proteome data, wrote the manuscript and critically revised the manuscript; CK performed Ig peptide preparation, processed the data and critically reviewed the manuscript for important intellectual content; MJ performed mass spectrometry processing and analysis and critically reviewed the manuscript for important intellectual content; FK performed mass spectrometry processing and analysis. SP reviewed the manuscript for important intellectual content UZ reviewed the manuscript for important intellectual content; SN analyzed the data and critically reviewed the manuscript for important intellectual content MK designed and supervised the study, treated the patients, collected and analyzed the data, performed statistical analysis, wrote the manuscript and critically revised the manuscript. All authors contributed to the article and approved the submitted version.
