# Supplementary material for: Cladribine treatment specifically affects peripheral blood memory B cell clones and clonal expansion in multiple sclerosis patients
Source: Front Immunol. 2023 Mar 7;14:1133967. doi: 10.3389/fimmu.2023.1133967 (PMC10028280; doi:10.3389/fimmu.2023.1133967)
Supplement: Supplementary file 1 [file DataSheet_1.pdf]

Supplementary data

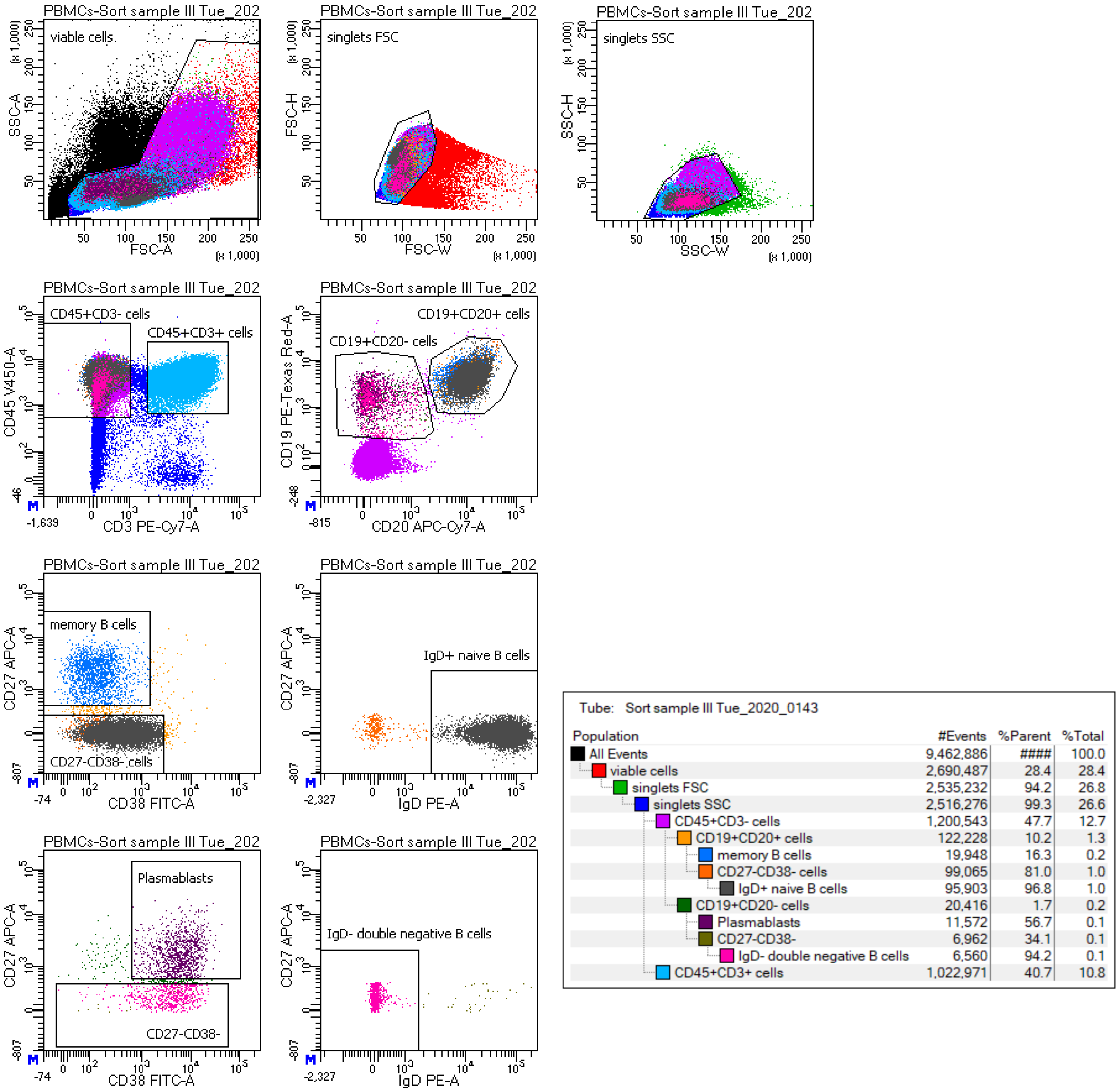

Tube: Sort sample III Tue_2020_0143

Population	#Events	%Parent	%Total
All Events	9,462,886	####	100.0
viable cells	2,690,487	28.4	28.4
singlets FSC	2,535,232	94.2	26.8
singlets SSC	2,516,276	99.3	26.6
CD45+CD3- cells	1,200,543	47.7	12.7
CD19+CD20+ cells	122,228	10.2	1.3
memory B cells	19,948	16.3	0.2
CD27-CD38- cells	99,065	81.0	1.0
IgD+ naive B cells	95,903	96.8	1.0
CD19+CD20- cells	20,416	1.7	0.2
Plasmablasts	11,572	56.7	0.1
CD27-CD38-	6,962	34.1	0.1
IgD- double negative B cells	6,560	94.2	0.1
CD45+CD3+ cells	1,022,971	40.7	10.8

Supplementary Figure S1: Exemplary gating strategy for flow cytometry sorting for different B cell subsets. B cell populations are defined as followed: naïve B cells CD19+CD20+CD27-CD38-/low IgD+, memory B cells CD19+ CD20+CD27+CD38low , double negative B cells (DN B cells) CD19+ CD20-/lowCD27-IgD-, plasmablasts CD19+CD20-/lowCD27+ CD38highIgD-)

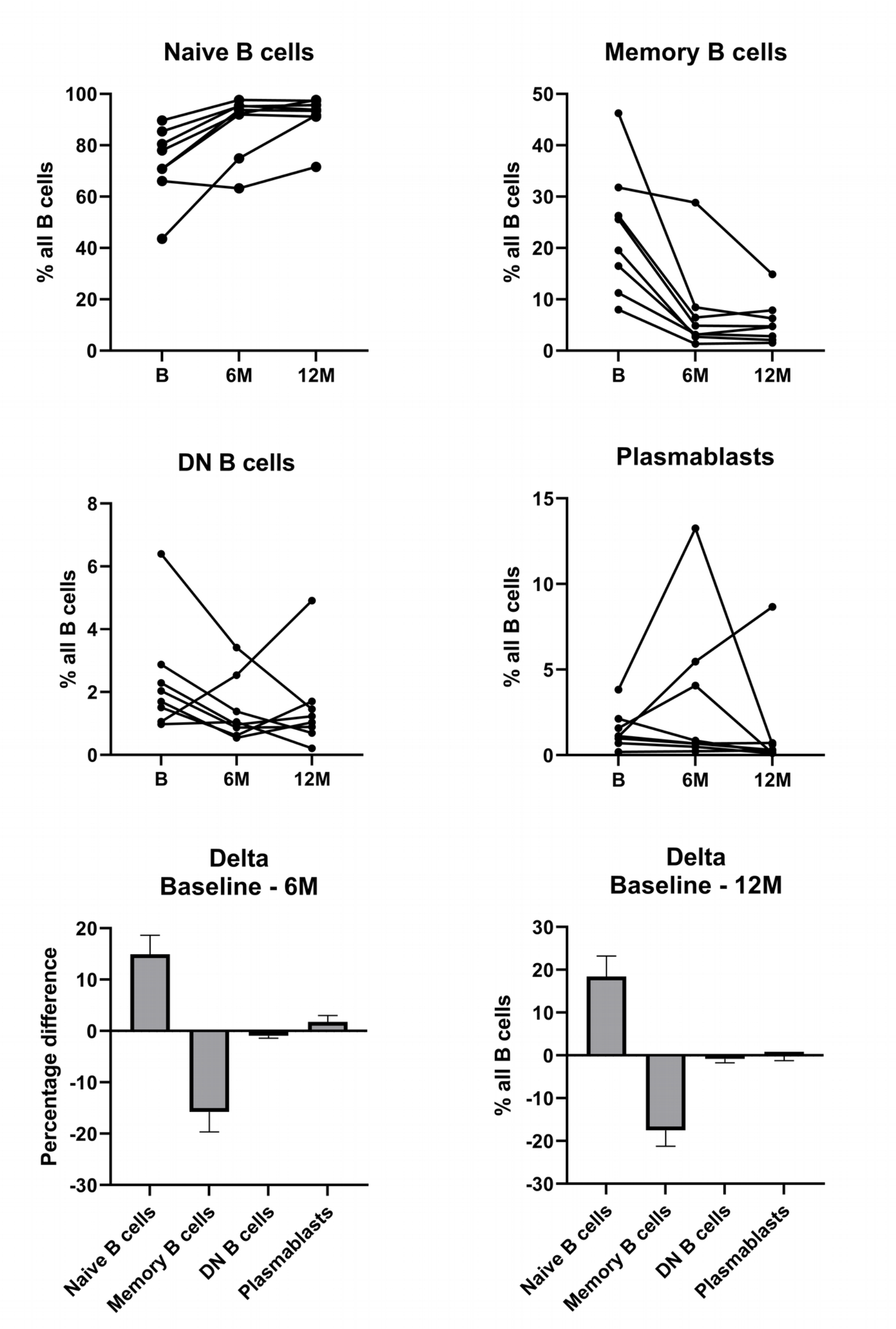

Supplementary Figure S2: Change in B cell sub-populations (naive B cells, memory B cells, double negative (DN) B cells and plasmablasts) for each individual subject and delta changes between baseline and 6 months (6M) and 12 months (12M).

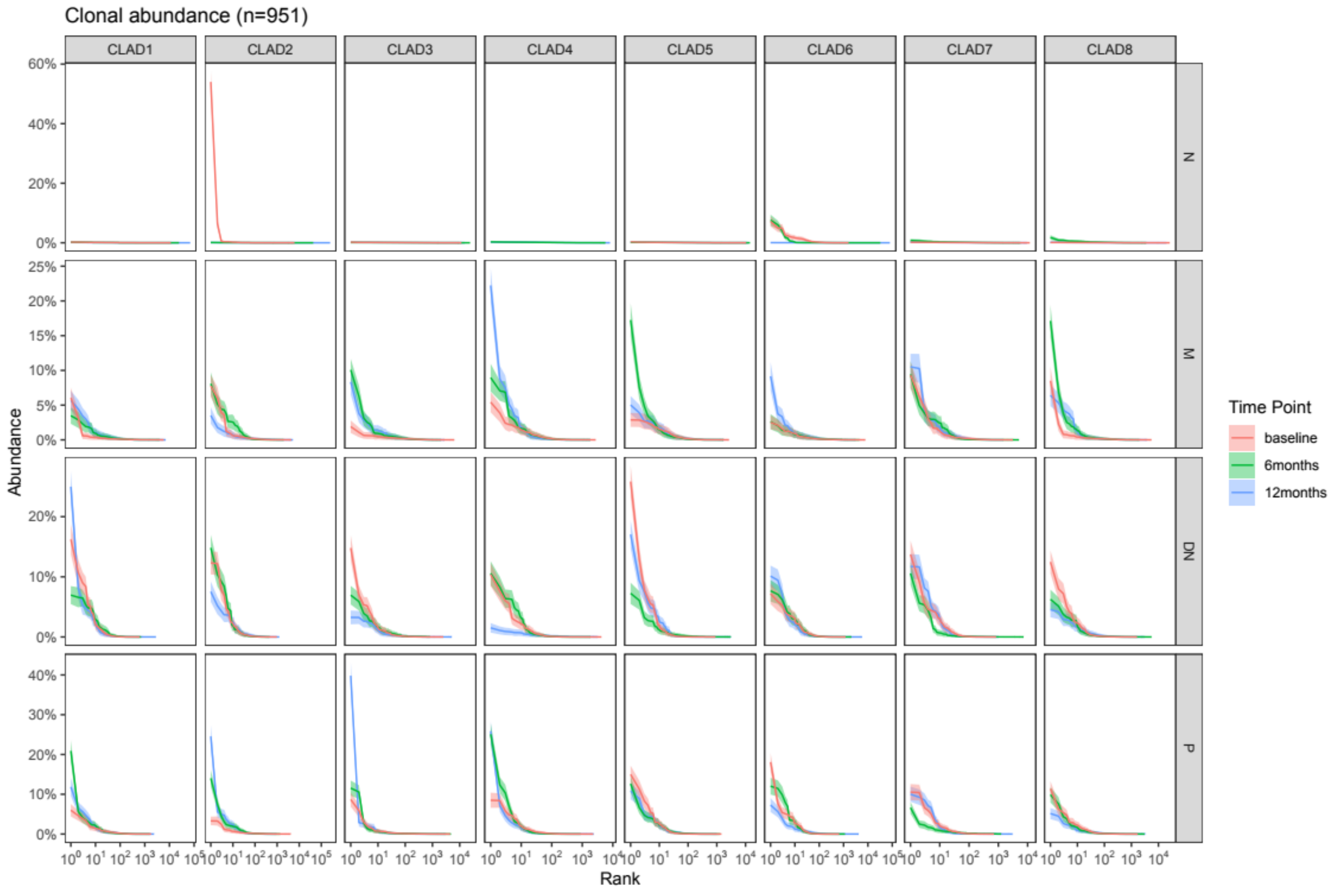

Supplementary Figure S3: Clonal abundance shown for each individual patient (CLAD1-8) and B cell population including naive (N), memory (M), double negative (DN) B cells and plasmablasts(P).The solid line shows the clonal abundance determined by bootstrapping sample of n = 951 sequences, with 200 repetitions. The shaded area shows the variation of the clonal abundance among the bootstrapping repetitions. Different time points (baseline, 6 months and 12 months following cladribine treatment) are colour coded.

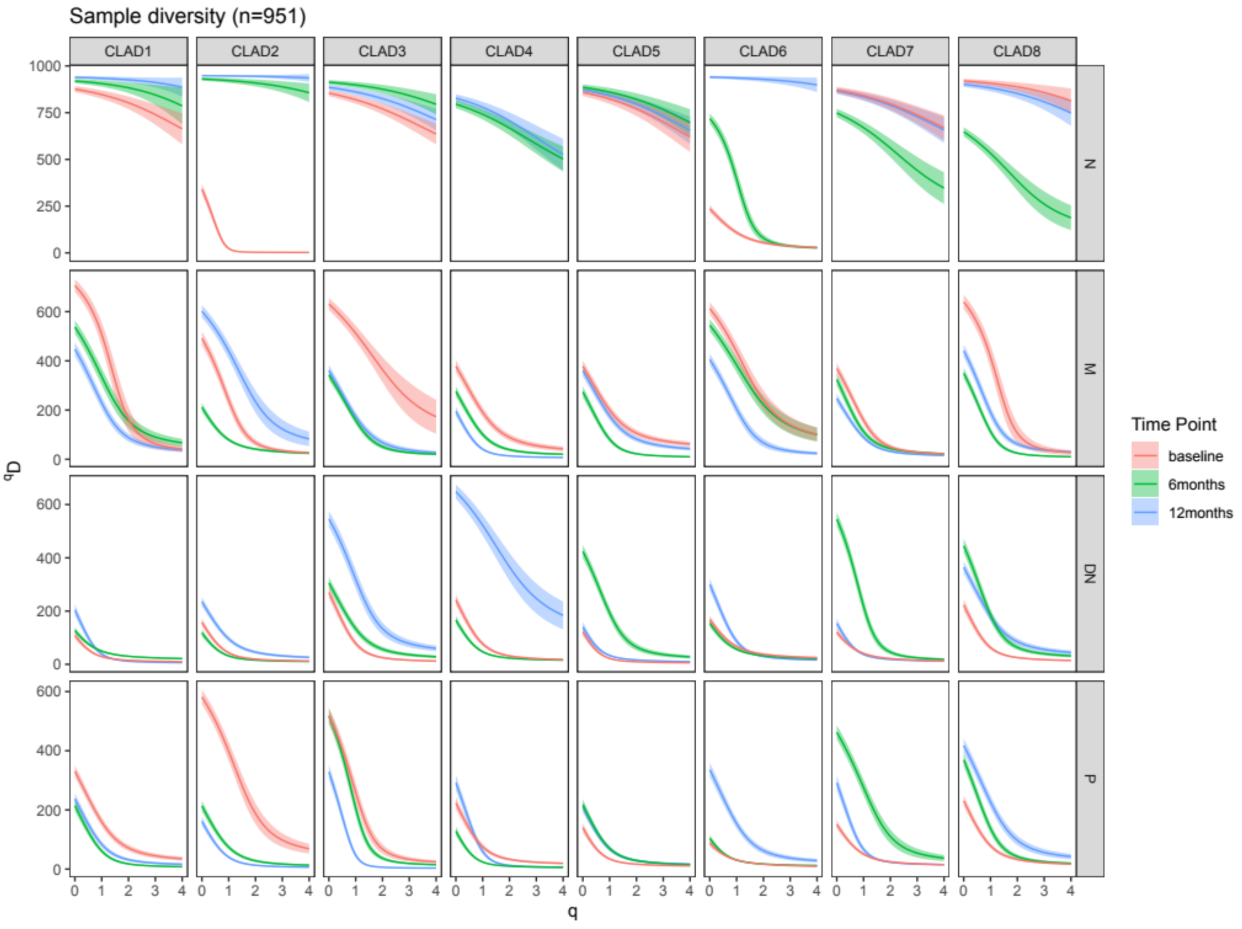

Supplementary Figure S4: Clonal diversity expressed as Hill Diversity curve shown for each individual patient (CLAD1-8) and B cell population including naive (N), memory (M), double negative (DN) B cells and plasmablasts (P). The solid line shows the clonal diversity determined by a bootstrapping sample of n = 951 sequences, with 200 repetitions. The shaded area shows the variation of the clonal diversity among the bootstrapping repetitions. Different time-points (baseline, 6 months and 12 months following cladribine treatment) are colour coded.

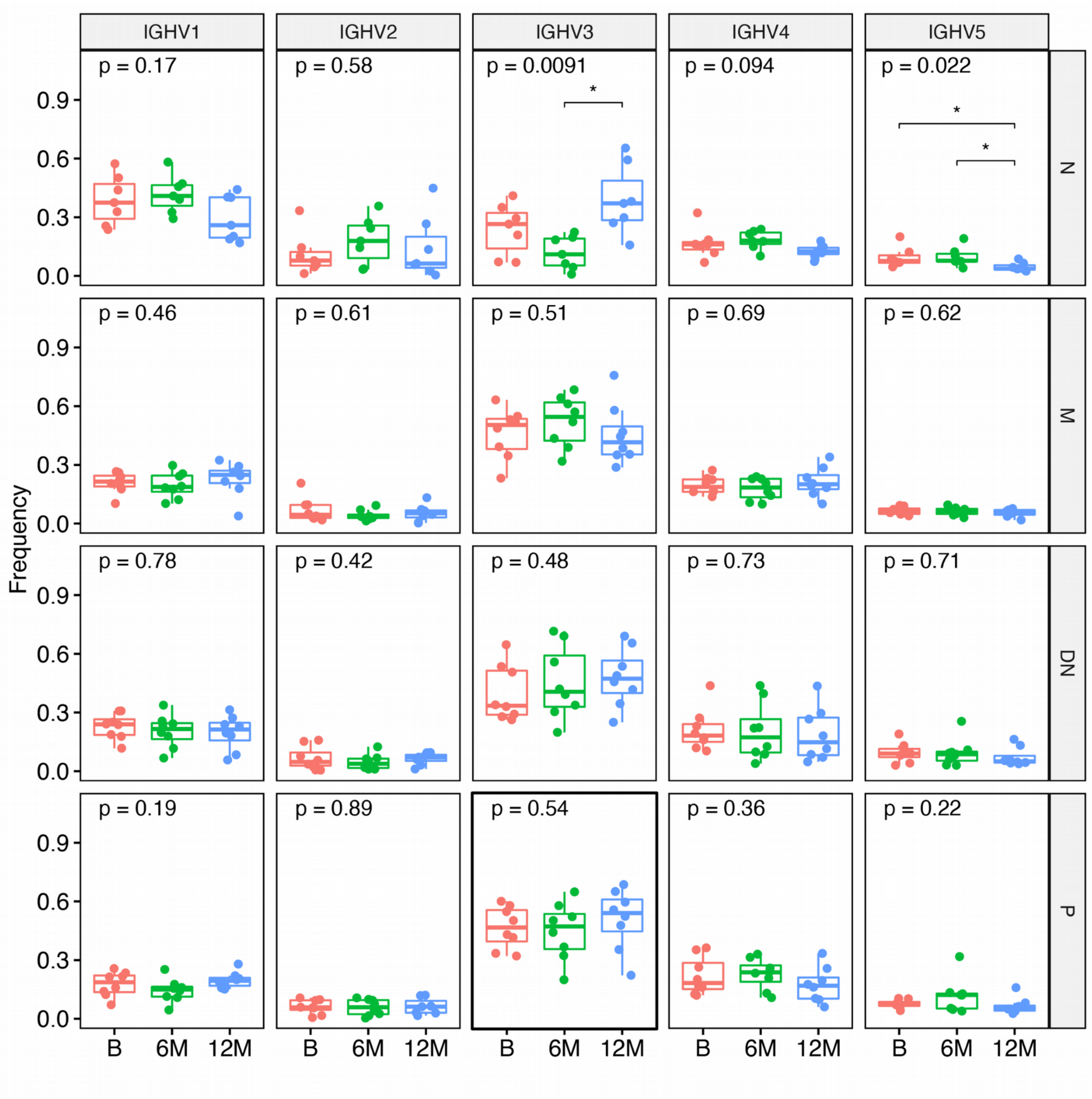

Supplementary Figure S5: IGHV germline distribution (IGHV1-5) for each B cell population including naive (N), memory (M), double negative (DN) B cells and plasmablasts(P) and time-point (baseline (B), 6 months (6M) and 12 months (12M) following cladribine treatment). P values show Kruskal-Wallis multi-group comparison tests. Asterisks denote Wilcoxon paired pairwise tests performed if Kruskal-Wallis tests were significant.

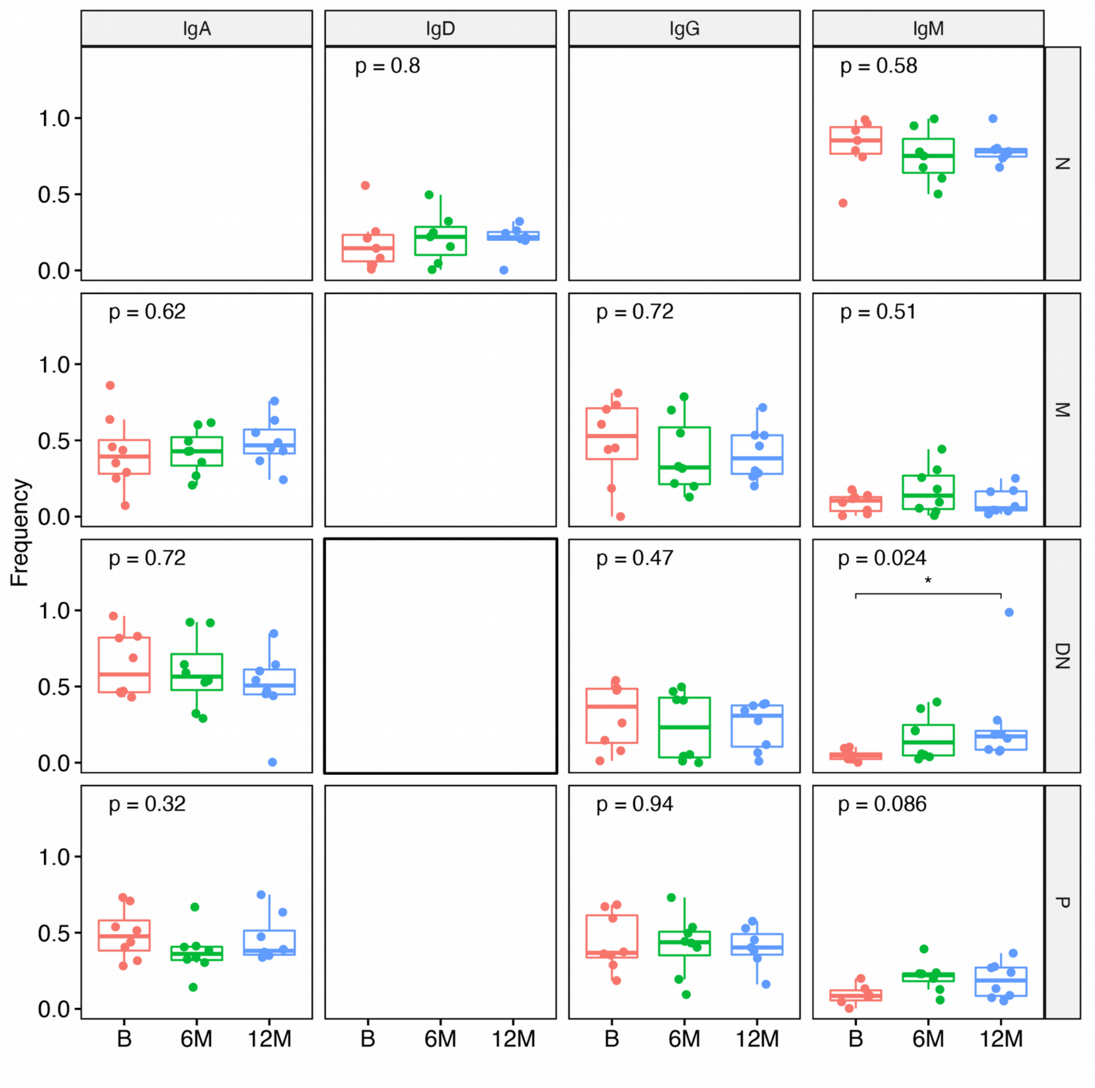

Supplementary Figure S6: Isotype percentages (IgA, IgD, IgG, IgM)) for each B cell population including naive (N), memory (M), double negative (DN) B cells and plasmablasts (P) and time-point (baseline (B), 6 months (6M) and 12 months (12M) following cladribine treatment). P values show Kruskal-Wallis multi-group comparison tests. Asterisks denote Wilcoxon Rank Sum paired tests performed if Kruskal-Wallis tests were significant. No primers were used for IgA, IgG in Naive B-cells, and no IgD primers were used for M, DN and P populations.

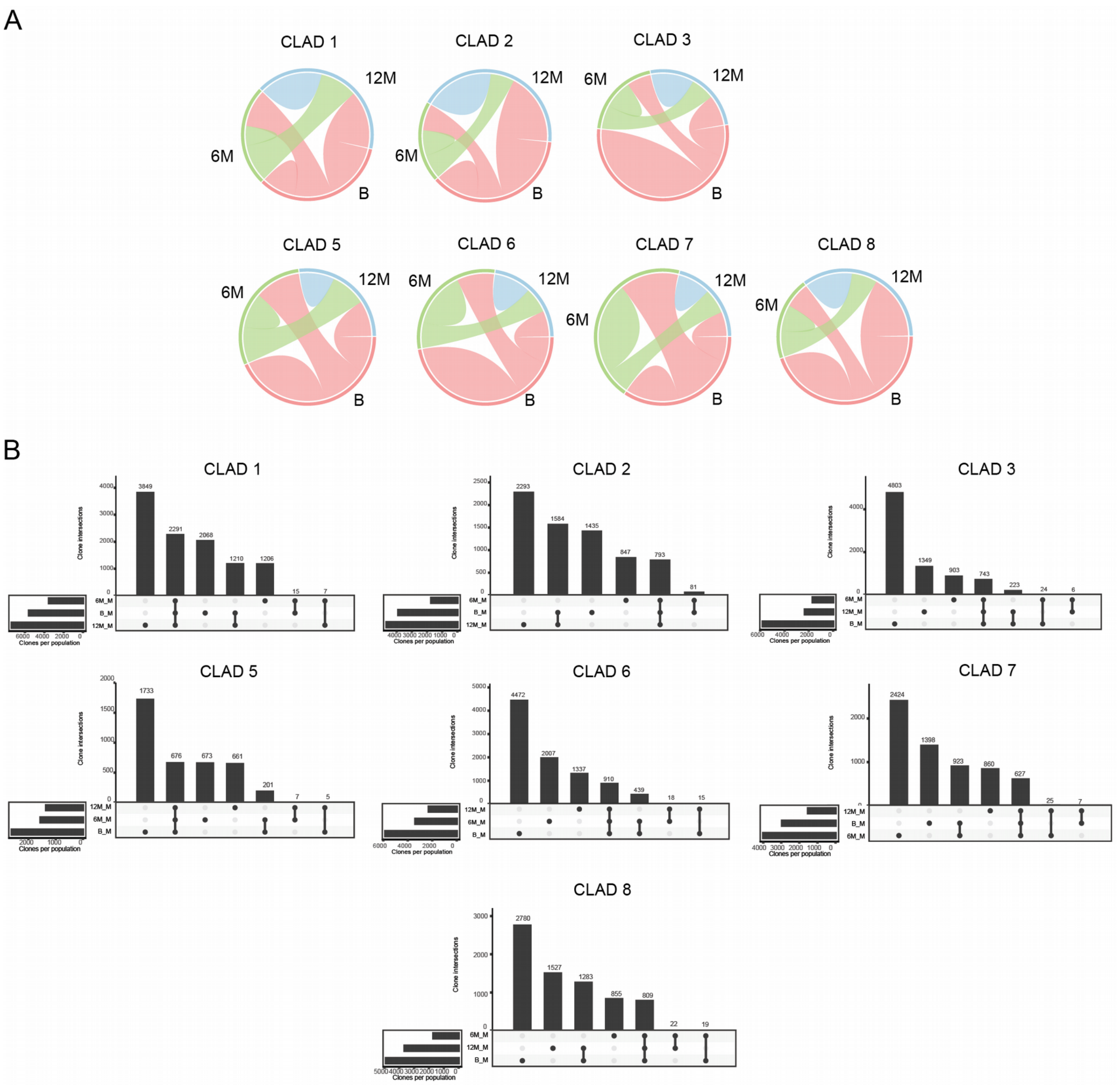

Supplementary Figure S7. Clonal overlap analyses among different time points for clones containing memory cells. A) Overlap plots show the proportion of clones comprising sequences that span over the different time points, and the proportion of clones comprising sequences that are only present in that time point. B) Set plots show the exact clonal overlap numbers (exclusive overlaps) among the three time points for each individual.

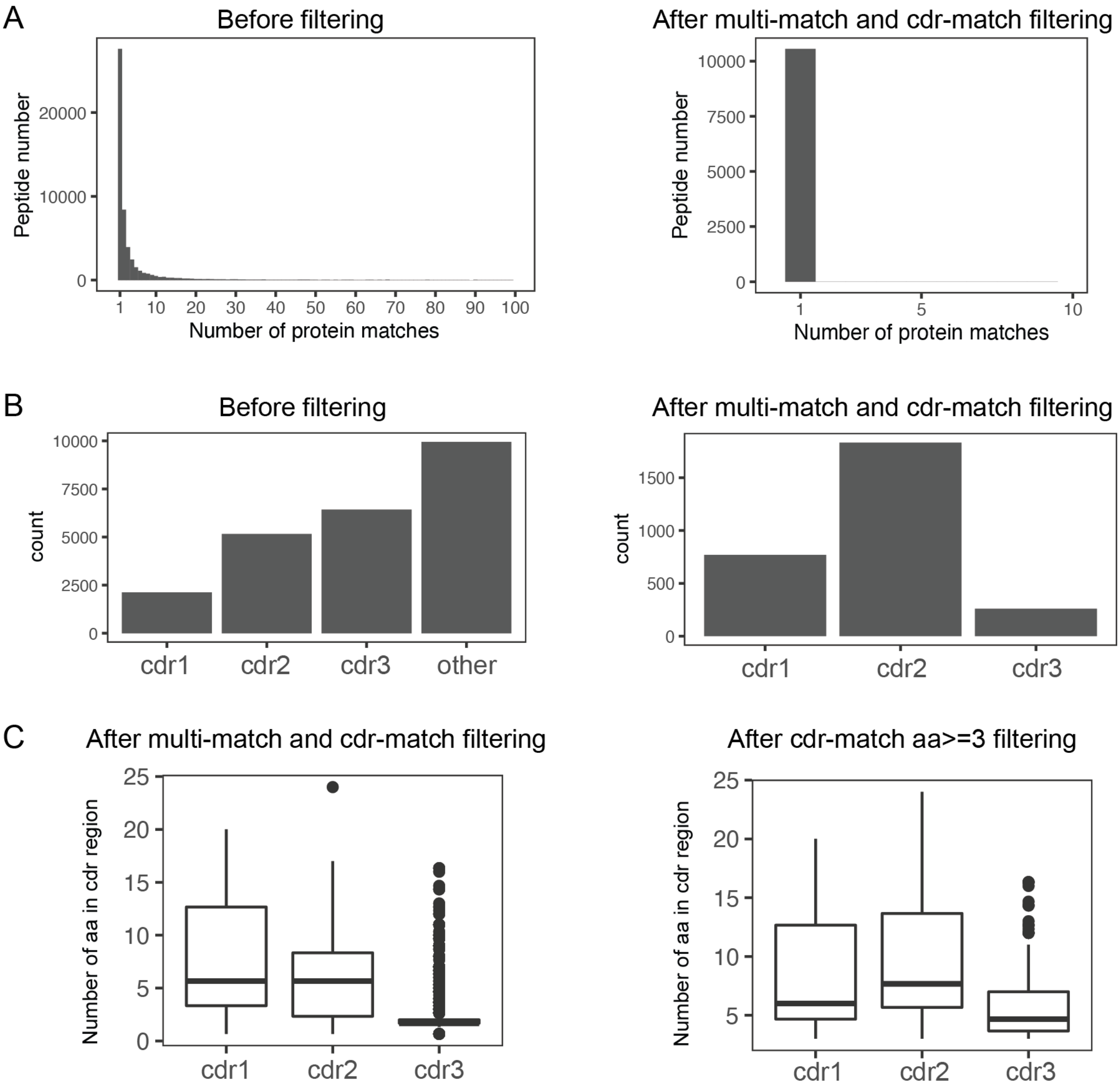

Supplementary Figure S8: Proteomics analysis peptide multi-match and cdr-match filtering procedure. Peptides were filtered out if they had ambiguous matches to the Ig transcriptome, or the matches did not align with at least 3 amino acids with the variable CDR regions. A) Effect of multi match filtering and CDR-match filtering on the peptide-Ig transcriptome match. Distribution of the number Ig transcriptome sequence matches per peptide before applying filtering (left). Distribution of the number of Ig transcriptome sequence matches after filtering for peptide-Ig transcriptome multimatches if those matches were not within the same B-cell clone. B) Number of peptides matching fully or partially to the CDR1, CDR2, CDR3 regions or other Ig transcriptome region, before peptide filtering (left). Number of peptides matching to the CDR regions after filtering out peptides that did not have at least 3 amino acids within those regions. C) Number of identified peptide amino acid (aa) matches within the cdr regions before cdr-match filtering (left). Number of peptide amino acid matches within the cdr regions after filtering for at least 3 amino acids matching within the cdr regions (right).
